# Decoding the Gut Microbiota in Multiple Sclerosis Using Nanopore Long-Read Sequencing: Insights into Disease Severity and Subtypes

**DOI:** 10.1007/s12035-025-05194-9

**Published:** 2025-07-18

**Authors:** Ameera Saeed Alshinnawy, Elham A. Badiea, Mahmoud Saad Swelam, Ahmed A. Sayed, Mohamed R. Mohamed

**Affiliations:** 1 Department of Biochemistry, Faculty of Science, Ain Shams University, Cairo, 11566 Egypt; 2 Department of Neurology, Faculty of Medicine, Ain Shams University, Cairo, Egypt; 3 Children’s Cancer Hospital 57357, Cairo, Egypt

**Keywords:** Gut microbiota, Nanopore, Long reads, Multiple sclerosis, Relapsing, Progressive

## Abstract

Multiple sclerosis (MS) is a chronic autoimmune disease of the central nervous system (CNS), characterized by neuroinflammation and neurodegeneration. Emerging evidence links gut microbiota dysbiosis to immune dysregulation and MS progression. While extensive research has been conducted in Western populations, region-specific studies are needed to assess the influence of local genetic and environmental factors. This study investigates gut microbiota alterations in Egyptian MS patients using Oxford Nanopore sequencing to identify microbial signatures associated with disease progression. Fecal samples from 33 newly diagnosed MS patients (20 with relapsing–remitting MS [RRM] and 13 with Progressive MS [PMS]) and 10 healthy controls were analyzed using long-read Oxford Nanopore sequencing of the full 16S rRNA gene. MS patients exhibited increased microbial richness but reduced evenness, with distinct gut microbiome profiles. Progressive MS was characterized by an abundance of pro-inflammatory bacteria (e.g., *Enterococcus faecium* and *Romboutsia timonensis*) and a depletion of short-chain fatty acid (SCFA)–producing species (*Ruminococcus bromii* and *Faecalibacterium duncaniae*), potentially contributing to heightened neuroinflammation and disease progression. Relapsing MS exhibited microbial shifts indicative of immune dysregulation, including increased *Clostridium saudiense* and decreased levels of the gut-protective *Faecalibacterium butyricigenerans*. Functional analysis linked these microbial alterations to oxidative stress, neurotransmitter imbalance, and suppressed lipid and carbohydrate metabolism. These findings underscore the role of gut microbiota dysbiosis in MS pathogenesis and, by focusing on an underexplored Egyptian cohort, highlight region-specific microbial shifts that may inform targeted therapeutic strategies for both Progressive and Relapsing forms of MS.

## Introduction

Multiple sclerosis (MS) is a chronic autoimmune disease of the central nervous system (CNS), characterized by demyelination, neuroinflammation, and neurodegeneration. It affects millions worldwide and is a leading cause of neurological disability among young and middle-aged adults, with women being approximately twice as likely to be affected as men [1]. MS is clinically categorized into three primary forms: relapsing–remitting MS (RRMS), primary progressive MS (PPMS), and secondary progressive MS (SPMS). Approximately 85% of patients are initially diagnosed with RRMS, characterized by episodes of acute attacks (relapses) followed by periods of remission. Among these patients, about two-thirds will eventually transition to SPMS, during which neurological disability accumulates independently of relapses [2]. Approximately 15% of patients present with PPMS, which is marked by continuous clinical deterioration from disease onset [3]. MS significantly impacts patients’ quality of life, family relationships, and occupational performance. This is particularly critical as MS often manifests during the peak years of family formation and employment. Early diagnosis and treatment are essential to prevent irreversible disability and to improve long-term outcomes [4].

The gut microbiota—a diverse community of microorganisms residing in the gastrointestinal tract—plays a vital role in maintaining immune homeostasis, metabolic function, and CNS health. Emerging evidence highlights the role of the gut-brain axis, a bidirectional communication network between the gut microbiota, the immune system, and the CNS, in the pathogenesis of MS. Dysbiosis, or an imbalance in gut microbial composition, has been implicated in promoting pro-inflammatory immune responses by shifting the balance from regulatory (Treg) to pro-inflammatory (Th1/Th17) T cells, thereby exacerbating CNS autoimmunity [5]. Mechanistically, dysbiosis can lead to disruption of the intestinal barrier—a multi-layered interface involving gut-associated immune tissue. This disruption increases intestinal permeability, commonly referred to as “leaky gut”, allowing microbial metabolites and inflammatory mediators to enter systemic circulation. These molecules can compromise the integrity of the blood–brain barrier (BBB), enabling peripheral immune cell infiltration into the CNS and triggering neuroinflammation [6]. In parallel, microbial signals also influence neuroinflammation and demyelination by modulating microglial activation. Several studies have reported distinct microbial signatures in MS patients compared to healthy controls [7]. Additionally, approximately 70% of MS patients exhibit increased intestinal permeability [8], which supports the hypothesis that altered gut barrier integrity may allow bacterial components such as microbial-associated molecular patterns to translocate, alter peripheral immune responses, or enter the CNS and drive autoimmunity [9].

Compositional changes in gut microbiota—especially reductions in beneficial short-chain fatty acid (SCFA)–producing bacteria such as *Faecalibacterium prausnitzii* and *Ruminococcus bromii* have—been widely documented in MS. These alterations are influenced by diet and nutritional status and are being targeted in clinical trials evaluating dietary interventions and fecal microbiota transplantation (FMT) [10].

Despite increasing evidence from Western populations, there is a lack of region-specific studies exploring gut microbiota alterations in MS patients from non-Western regions such as the Middle East. The rising incidence of MS in countries like Egypt necessitates a better understanding of local environmental, genetic, and dietary factors that may shape gut microbial composition and influence disease mechanisms [11].

Existing studies have primarily focused on Western cohorts and short-read sequencing technologies, which often restrict taxonomic resolution to the genus level and overlook regional microbial signatures influenced by distinct dietary and environmental exposures. Furthermore, functional predictions derived from gut microbiota composition have rarely been explored in Middle Eastern MS cohorts.

To address these gaps, this study investigates gut microbiota alterations in Egyptian MS patients using Oxford Nanopore long-read sequencing, which enables full-length 16S rRNA gene analysis and thereby improves species-level taxonomic resolution and downstream functional predictions. While certain bacterial species share highly similar or even identical 16S rRNA sequences, limiting their discriminability using this marker alone [12, 13], full-length sequencing using Oxford Nanopore has been shown to significantly enhance species-level classification when combined with robust bioinformatic tools and curated reference databases [14, 15]. Thus, despite some inherent limitations, this approach offers a more comprehensive and confident characterization of gut microbial communities in this understudied population.

We hypothesize that region-specific microbiome shifts contribute to MS pathogenesis through altered microbial functions and pro-inflammatory mechanisms.

## Materials and Methods

### Sample Collections and Study Design

This study was approved by the Research Ethics Committee (REC) at the Faculty of Medicine, Neurology and Psychiatry Department, Ain Shams University, Egypt (FMASU MD 188/2024); written informed consent was obtained from all participants. All procedures were conducted in accordance with relevant guidelines and regulations. Ethnicity-matched healthy controls were also recruited, with approximately half being family members of MS patients. This approach was used intentionally to help mimic shared lifestyle and environmental exposures, particularly dietary habits and living conditions, thus minimizing lifestyle-related confounding variables. Participants were recruited from the Multiple Sclerosis Unit, Neurology Department, Faculty of Medicine, Ain Shams University Hospitals, Cairo, Egypt, between September 2024 and January 2025.

A total of 33 newly diagnosed MS patients, including 20 with Relapsing MS and 13 with Progressive MS, and 10 age- and sex-matched healthy volunteers were included as controls. All participants were residents of Greater Cairo and the surrounding Nile Delta region, ensuring geographic consistency.

MS diagnosis was established based on the 2017 revised McDonald criteria, integrating clinical assessment, magnetic resonance imaging (MRI), and cerebrospinal fluid (CSF) analysis when necessary. Exclusion criteria included the following: current or previous use of disease-modifying or biologic therapies for MS, use of antibiotics within the past 3 months, diagnosed inflammatory bowel disease, any gastrointestinal tract surgery, or an active malignancy. Participants were also excluded if they had special or restrictive diets, recent gastrointestinal infections, or were taking probiotic or prebiotic supplements. All participants followed their usual dietary patterns during the study period, and no dietary interventions were applied. Patients with obesity or diabetes were also excluded. All participants underwent a comprehensive medical and neurological assessment, including collection of demographic and clinical data, Expanded Disability Status Scale (EDSS) scores, and evaluation of MS disease activity. Additionally, all MS patients underwent brain magnetic resonance imaging (MRI), and diagnosis was confirmed based on clinical neurological manifestations, radiological evidence of characteristic MS lesions, and/or cerebrospinal fluid (CSF) analysis indicating the presence of MS-associated antibodies.

### Sample Processing and DNA Extraction

Fecal samples were collected in the morning, within the same time window (7:00–10:00 AM) to minimize diurnal variation in gut microbiota into sterile Cary-Blair transport tubes using a microbiological swab from the Fecal Swab Sampling Kit and stored at − 80 °C. In cases where stool sample collection was not feasible, rectal swabs were used. Genomic DNA was extracted using the DNeasy PowerSoil Pro Kit (QIAGEN, Germany) according to the manufacturer’s protocol. The quality and concentration of extracted DNA were assessed using a NanoDrop 2000 spectrophotometer (Thermo Fisher Scientific, USA).

### 16S rRNA Gene PCR Amplification and Library Preparation

#### ONT MinION 16S V1–V9 Library Preparation

Library preparation was performed using the Oxford Nanopore Technologies 16S Barcoding Kit (SQK-16S024), which enables amplification and barcoding of the full 16S rRNA gene (V1–V9) region. PCR amplification of the full 16S hypervariable region (V1–V9) [12] was performed using the 27 F forward primer (5′-ATCGCCTACCGTGAC–barcode–AGAGTTTGATCMTGGCTCAG–3′) and the 1492R reverse primer (5′–ATCGCCTACCGTGAC–barcode–CGGTTACCTTGTTACGACTT–3′), both containing 5′ tags to facilitate ligase-free adapter attachment. Each 10 ng DNA sample was amplified using the 16S Barcoding Kit 0–24 under the following conditions: initial 15 min denaturation at 98 °C (Stage 1), 25 cycles of 20 s denaturation at 98 °C, 30 s annealing at 55 °C, 2 min extension at 65 °C (Stage 2), and 5 min final extension at 65 °C (Stage 3), longAmp Hot Start Taq 2 × Master Mix. The 16S V1–V9 amplicons were subsequently purified using AMPure XP magnetic beads with a PCR reaction mix to magnetic bead ratio of 5:3 and washed twice with freshly prepared 80% ethanol. The final elution of purified DNA was performed by adding 10 µL of 10 mM Tris–HCl pH 8.0 with 50 mM NaCl, incubating for 2 min at room temperature, and recovering 10 µL of the elute from each tube. DNA concentration of the purified 16S V1–V9 amplicons was measured using a Qubit 4 fluorometer (Thermo Fisher Scientific, USA), and samples were pooled to a total of 40 ng in 10 µL for library preparation.

#### 16S rRNA Gene Amplicon Sequencing

Sequencing was performed using the MinION Flow Cell (Oxford Nanopore Technologies, UK) and MinKNOW software (Oxford Nanopore Technologies, UK; v21.06.13). FAST5 raw data were collected over 48 h per run and basecalled into FASTQ files using the GPU-based Guppy basecaller (Oxford Nanopore Technologies, UK; v5.0.16) in super-accurate mode, with barcode and adapter trimming enabled. Sequencing continued until the barcode with the fewest reads exceeded 100,000 passed reads, ensuring sufficient depth across all samples. While MinION Flow Cells can generate high sequencing throughput, the number of samples per run was limited to 24 due to the barcode limit of the SQK-16S024 kit. Therefore, each flow cell was used to multiplex up to 24 samples, in accordance with the kit’s design specifications.

### Bioinformatics and Statistical Analysis

For each sample, high-quality reads from the/pass subdirectory, as generated by the Guppy basecaller, were concatenated into a single FASTQ file. These files were then assessed for quality using FastQC (v0.11.9). FASTQ files that met quality thresholds were then merged into a single file for each individual sample, ensuring all sequencing reads belonging to a participant were consolidated for downstream analysis. Adapter sequences were trimmed using Porechop_ABI (v. 0.5.5) (https://github.com/rrwick/Porechop_ABI), and low-quality, short, or long reads were filtered with NanoFilt (v.2.8.0) with a minimum read length of 500 bp and a minimum average quality score of Q10 [16]. Cleaned reads were analyzed using the Epi2me Metagenomics Pipeline (Oxford Nanopore Technologies, UK), aligning reads to the NCBI RefSeq 16S–18S database with minimap2 (v. 2.24-r1122)[17], samtools (v. 1.16.1)[18], and taxonkit (v. 0.14.1) to assign taxonomy and read counts per sample [19]. Prior to downstream analysis, low-abundance taxa were filtered by excluding features with a total read count less than 10 and relative abundance of prevalent microbiota (> 1% in any sample group) to minimize the impact of spurious or rare taxa.

Downstream analysis and visualization were conducted using R (v4.4.2) and the following R packages: Heatmap Construction: Heatmaps were generated using the ComplexHeatmap package (v2.18.0)[20]. Hierarchical clustering was based on Bray–Curtis dissimilarity and complete linkage method. Diversity Analysis: Alpha diversity (α-diversity) metrics (Observed OTUs, Richness, Evenness) were calculated using the phyloseq package (v1.44.0)[21]. Beta diversity (β-diversity) was assessed using vegan (v2.6–4), with Bray–Curtis dissimilarity and weighted/unweighted UniFrac distances computed via phyloseq. Raw *p*‐values from PERMANOVA tests (Bray–Curtis dissimilarities) were adjusted using the Benjamini–Hochberg FDR procedure; features with FDR‐adjusted *q* < 0.05 were considered statistically significant. Non-metric multidimensional scaling (NMDS) and principal coordinate analysis (PCoA) plots were generated using vegan and phyloseq. Visualization: all bar plots, box plots, and diversity plots were created using the ggplot2 package (v3.4.2). Statistical testing: normality of data was assessed using the Shapiro–Wilk test. α-Diversity comparisons between groups were evaluated using either *t*-test (parametric) or Wilcoxon rank-sum test (non-parametric), based on the normality outcome. β-Diversity differences were tested using PERMANOVA via the adonis2 function in vegan. Differential abundance testing was conducted at the species level using the DESeq2 package (v1.42.0). Count data were pre-filtered to exclude taxa with fewer than 10 reads across all samples. Functional prediction and analysis: functional predictions were generated using PICRUSt2 (v2.5.2)[22]. KEGG Orthology (KO) and MetaCyc pathway-level abundances were analyzed with the ALDEx2 package for differential enrichment, applying clr-transformation and Wilcoxon tests for comparison. Correlation analysis: Spearman’s rank correlation was used to evaluate associations between bacterial abundances and clinical metadata (e.g., EDSS and total relapses) using the psych R package (v2.3.6). Correlation matrices were visualized using corrplot package (v0.92). Data with normal distribution were reported as mean ± standard deviation (SD), while non-normally distributed data were expressed as median ± standard error (SE). Correlation analyses were performed using Spearman’s rank correlation coefficient with significance set at *p* < 0.05.

**Fig. 1:**
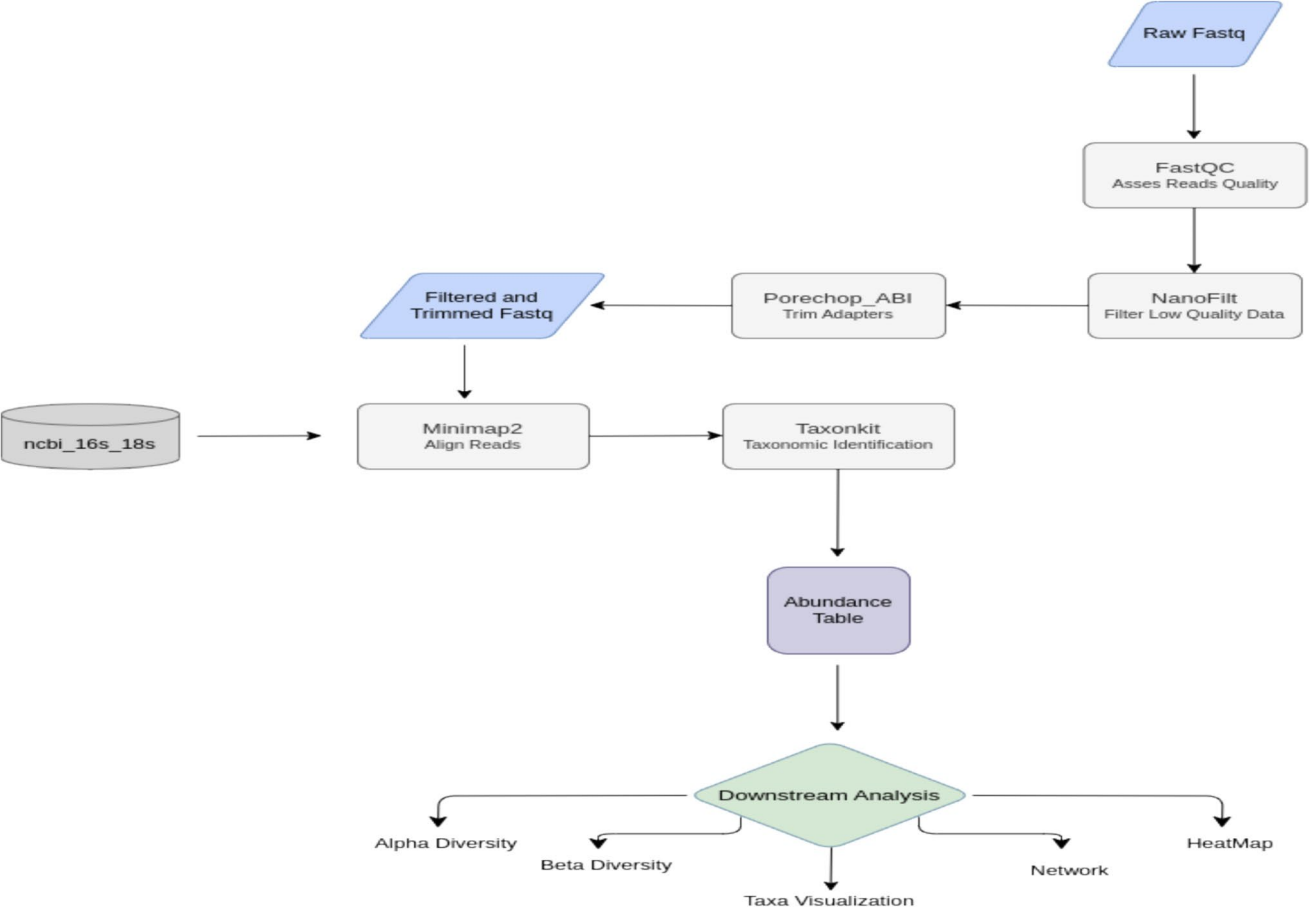
Overview of the bioinformatics workflow used for processing and analyzing full-length 16S rRNA gene sequencing data in this study

An overview of the bioinformatics workflow used to process and analyze the full-length 16S rRNA gene sequences is shown in Fig. 1. To ensure sufficient sequencing depth, 100,000 reads per sample were retained for taxonomic analysis. α-Rarefaction curves were used to verify diversity saturation, and β-diversity was assessed using Bray–Curtis dissimilarity and weighted/unweighted UniFrac distances [23].

## Results

**Fig. 2:**
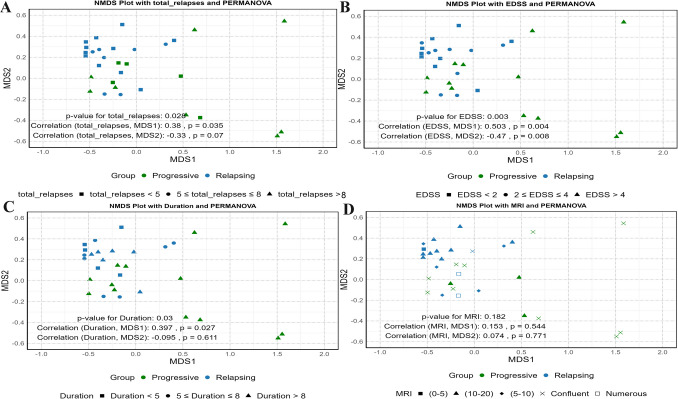
NMDS (non-metric multidimensional scaling) plots illustrating gut microbiota community structure in MS patients in relation to clinical parameters, based on Bray–Curtis dissimilarity. **A** Microbiota composition by total relapse frequency: squares (< 5), circles (5–8), triangles (> 8). PERMANOVA *p* = 0.028; correlation with NMDS1 = 0.38 (*p* = 0.035), NMDS2 = − 0.33 (*p* = 0.07). **B** Microbiota profiles by EDSS categories: squares (< 2), circles (2–4), triangles (> 4). PERMANOVA *p* = 0.003; NMDS1 correlation = 0.503 (*p* = 0.004), NMDS2 = − 0.47 (*p* = 0.008). **C** Disease duration groups: squares (< 5 years), circles (5–8 years), triangles (> 8 years). PERMANOVA *p* = 0.03; NMDS1 correlation = 0.397 (*p* = 0.027), NMDS2 = − 0.095 (*p* = 0.611). **D** MRI lesion load: squares (0–5), circles (5–10), triangles (10–20), X (confluent), white square (numerous). PERMANOVA *p* = 0.182; NMDS1 correlation = 0.153 (*p* = 0.544), NMDS2 = 0.074 (*p* = 0.771). All analyses were performed using Bray–Curtis dissimilarity and visualized with NMDS ordination. Disease type is consistently represented by color: green (Progressive MS) and blue (Relapsing MS)

Fecal samples were collected from 33 MS patients and 10 healthy controls; the average age of patients with Relapsing MS was 31.7 years, while that of patients with Progressive MS was 38 years, and 67% were women. Over half of the patients were retired due to clinical deterioration, while 40% were still in employment; details of the study population are provided in Table 1. The EDSS scores of MS patients were 2.25 ± 0.77 (median ± SE) for the Relapsing MS group and 5.65 ± 0.60 (mean ± SD) for the Progressive MS group, based on the respective data distributions. Disease duration ranged from 1 to 14 years (Fig. 2). Figure 2 summarizes the NMDS ordination of gut microbiota structure in relation to clinical characteristics of MS patients, including total relapses (Fig. 2A), EDSS scores (Fig. 2B), disease duration (Fig. 2C), and MRI lesion load (Fig. 2D). These plots illustrate microbial variation based on Bray–Curtis dissimilarity, with PERMANOVA and correlation results shown for each parameter.

**Table 1:** Demographic and clinical data of the study population

Parameter	Control (*n* = 10)	Relapsing MS (*n* = 20)	Progressive MS (*n* = 13)
Age (years)	33.8 ± 5.51	31.7 ± 5.86	38.0 ± 4.15
BMI (kg/m^2^)	24.64 ± 3.34	25.93 ± 2.33	26.50 ± 1.67
Male *n* (%)	4(40%)	5(25%)	6(46%)
Female *n* (%)	6(60%)	15(75%)	7(54%)
EDSS score	NA	2.25 ± 0.77	5.65 ± 0.60

Table 1 presents the demographic and clinical characteristics of the study participants, including age, body mass index (BMI), gender distribution, and EDSS score. Age and BMI are reported as mean ± standard deviation, while gender distribution is expressed as the percentage of males and females in each group. The table compares the control group (*n* = 10) with the MS subgroups: Relapsing (*n* = 20) and Progressive (*n* = 13).

The NMDS analysis reveals significant associations between gut microbial community structure and clinical parameters in MS patients. Specifically, microbial composition varies notably with total relapse frequency (PERMANOVA *p* = 0.028), EDSS score (*p* = 0.002), and disease duration (*p* = 0.03), highlighting the influence of disease severity on β-diversity. Samples from progressive MS patients—characterized by higher EDSS scores, longer disease duration, and increased relapse counts—demonstrate greater dispersion along NMDS axes, suggesting enhanced microbial community divergence or dysbiosis with advancing disease. In contrast, no significant correlation was found between microbiota β-diversity and MRI lesion load (*p* = 0.174), indicating that lesion burden may not directly relate to gut microbial shifts in this cohort.

### Gut Microbiota α and β-Diversity

**Fig. 3:**
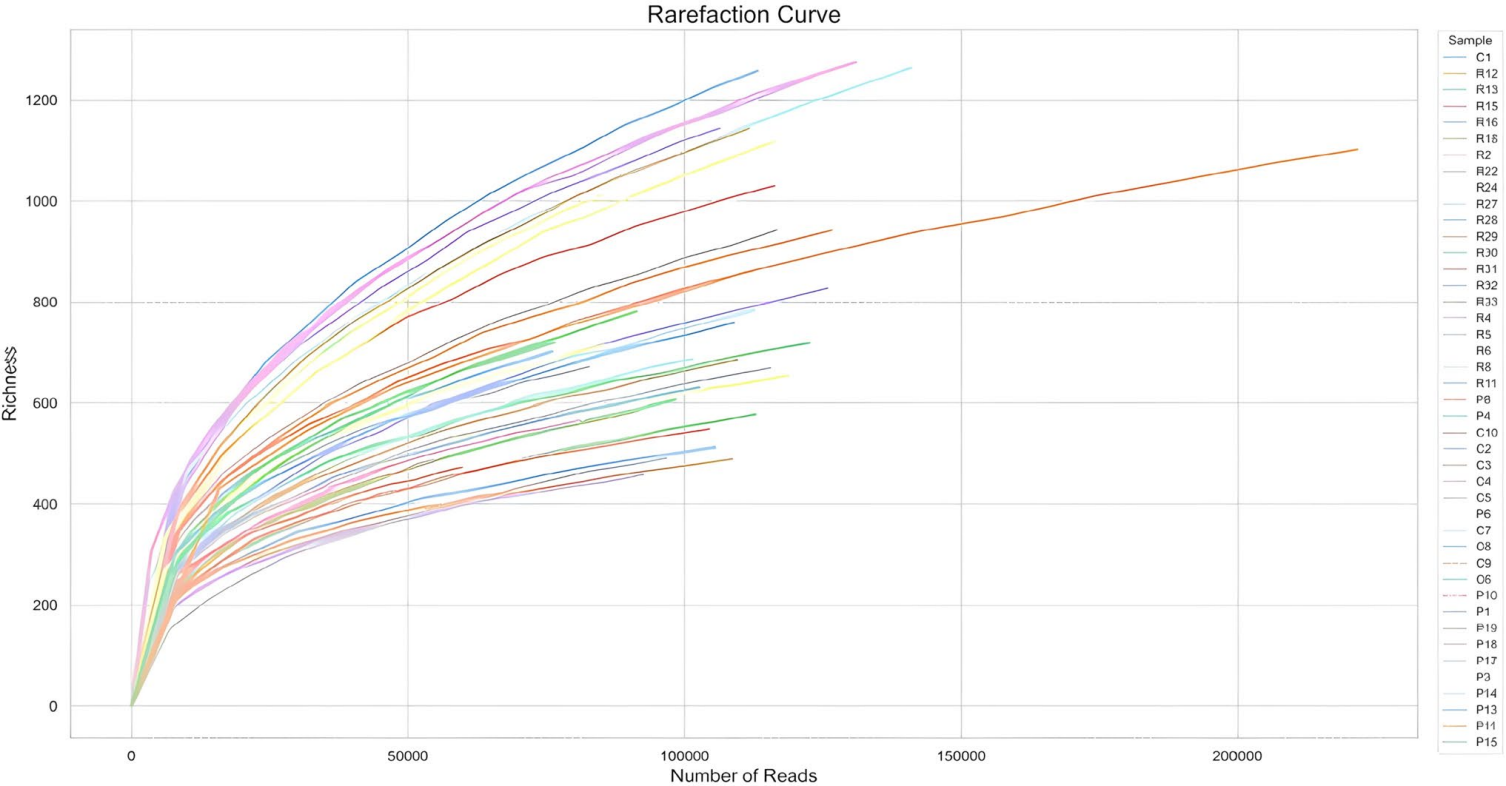
Rarefaction curve of gut microbiota from MS and Control group. Sample-based rarefaction curves displaying observed species richness. Sample size indicates the number of reads sampled from the total amount of reads analyzed during the real-time analysis. The Y-axis represents the number of unique bacterial species identified at the species-level taxonomic resolution in those subsampled reads

To assess overall differences in microbial communities between MS patients and healthy controls, we calculated measures of alpha and β-diversity. α-Diversity, reflecting species richness and evenness, was evaluated at multiple sequencing depths using rarefaction curves. The rarefaction curves for all samples reached a plateau, indicating that the sequencing effort was sufficient to capture the majority of species present. This suggests that additional sequencing would likely provide minimal new information (Fig. 3).

**Fig. 4:**
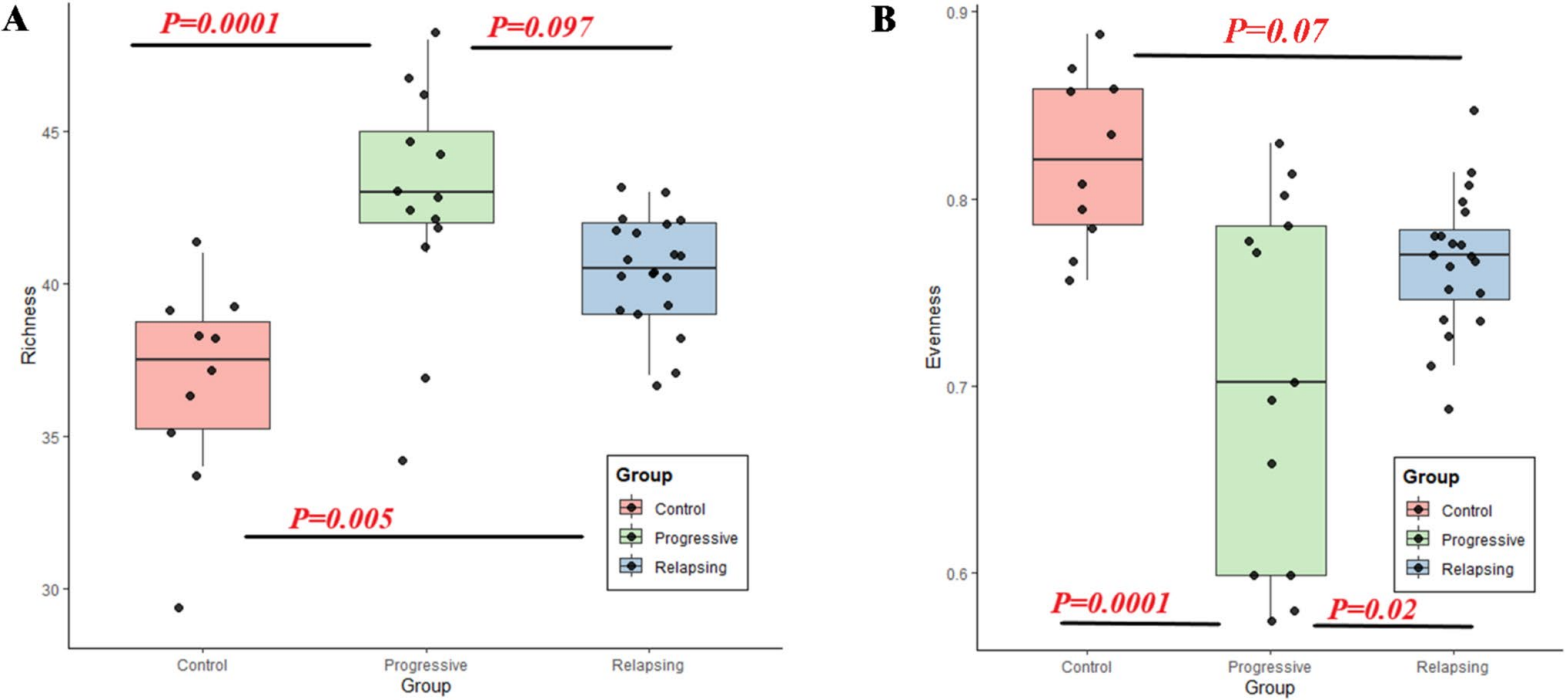
Box plot of microbial α-diversity estimation in MS groups compared to control **A** Richness diversity index. **B** Evenness diversity index, reflecting the distribution of species abundance within each group. Statistical comparisons were performed to evaluate differences among groups

The overall species richness of the gut microbiota in the Relapsing group was significantly different from that of the Control group (*P* = 0.005 for observed OTU number). Similarly, the Progressive group also showed a significant difference from the Control group (*P* = 0.0001). However, there was no significant difference between the Relapsing and Progressive groups (*P* = 0.097) (Fig. 4A). The Control group consistently exhibited higher evenness, indicating a more balanced distribution of species, whereas both the Progressive and Relapsing groups demonstrated reduced evenness. These findings suggest that MS alters community structure and species distribution.

The evenness diversity index revealed a statistically significant difference between the Progressive and Control groups (*P* = 0.0001), indicating that microbial species in the Progressive group are less evenly distributed. A significant difference was also observed between the Relapsing and Progressive groups (*P* = 0.02), whereas no significant difference was found between the Control and Relapsing groups (*P* = 0.07) (Fig. 4B).

**Fig. 5:**
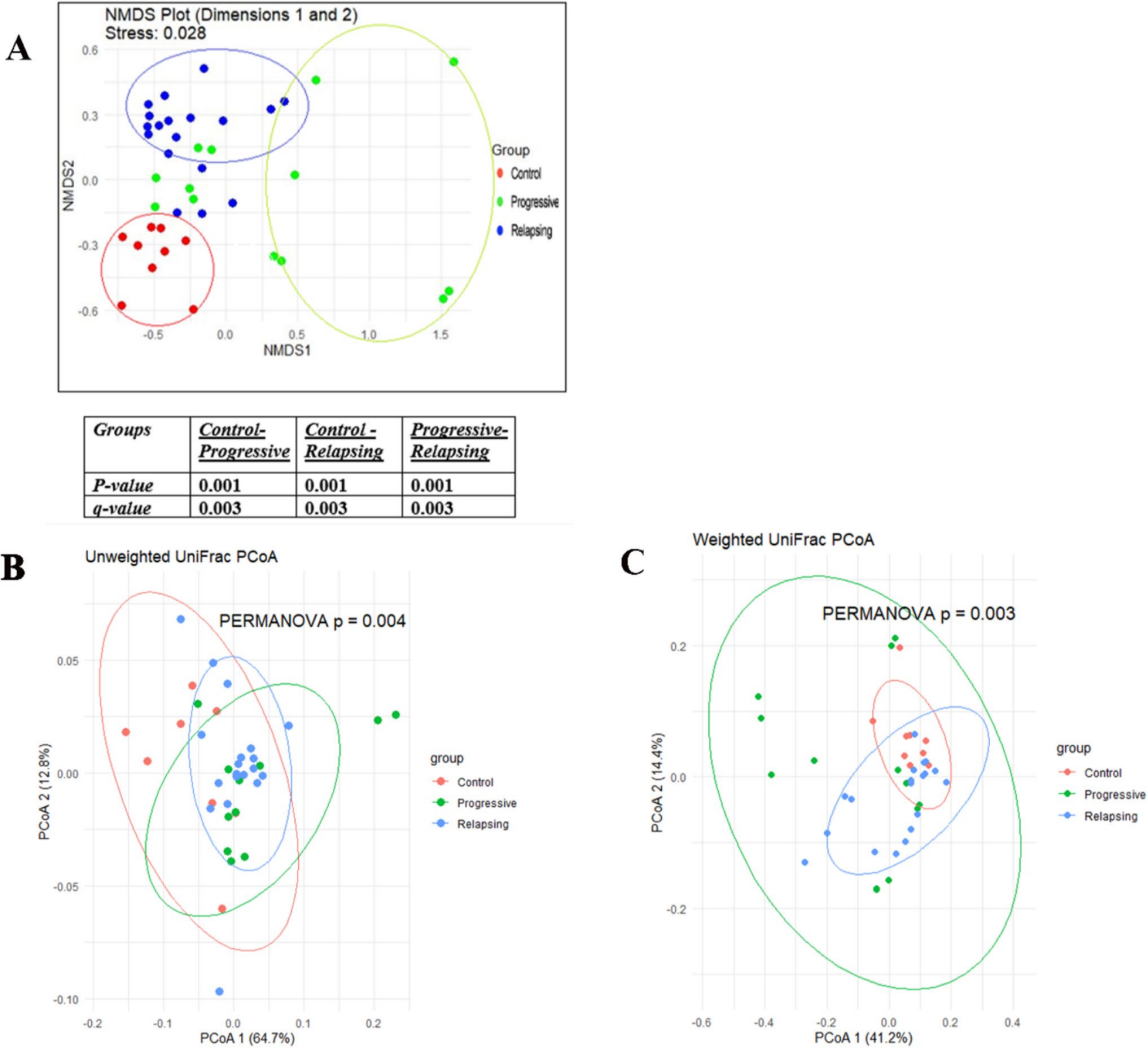
**A** Non-metric multidimensional scaling (NMDS) plots of microbial communities. PERMANOVA analysis with false discovery rate (FDR) adjusted *P* value. **B** Difference analysis of β-diversity of gut microbiota in the Control group and MS groups PCoA analysis based on unweighted UniFrac distance. **C** PCoA analysis based on weighted UniFrac distance

β-Diversity measures the compositional dissimilarity or variation between microbial communities across different groups. The NMDS plot (Fig. 5A) shows distinct clustering for each group, with some overlap—particularly between the Progressive and Relapsing groups. The stress value of 0.028 indicates an excellent fit for the NMDS model, meaning the plot accurately preserves the dissimilarities or distances between the samples. PERMANOVA analysis revealed significant differences in microbial community composition across all pairwise group comparisons: Control vs. Progressive, Control vs. Relapsing, and Progressive vs. Relapsing. Additionally, principal coordinates analysis (PCoA) based on both unweighted and weighted UniFrac distances demonstrated significant differences in microbiota distribution among the groups (Fig. 5B, C).

### MS-Associated Microbiota Changes at the Genus and Species Level

The relative abundance of microbial species was analyzed across healthy individuals (Control) and MS groups (Progressive and Relapsing). Overall, MS groups exhibited distinct compositional differences from the Control group. Notably, the Control group was enriched in health-associated taxa, while MS groups showed increased prevalence of pro-inflammatory and dysbiosis-associated species.

**Fig. 6:**
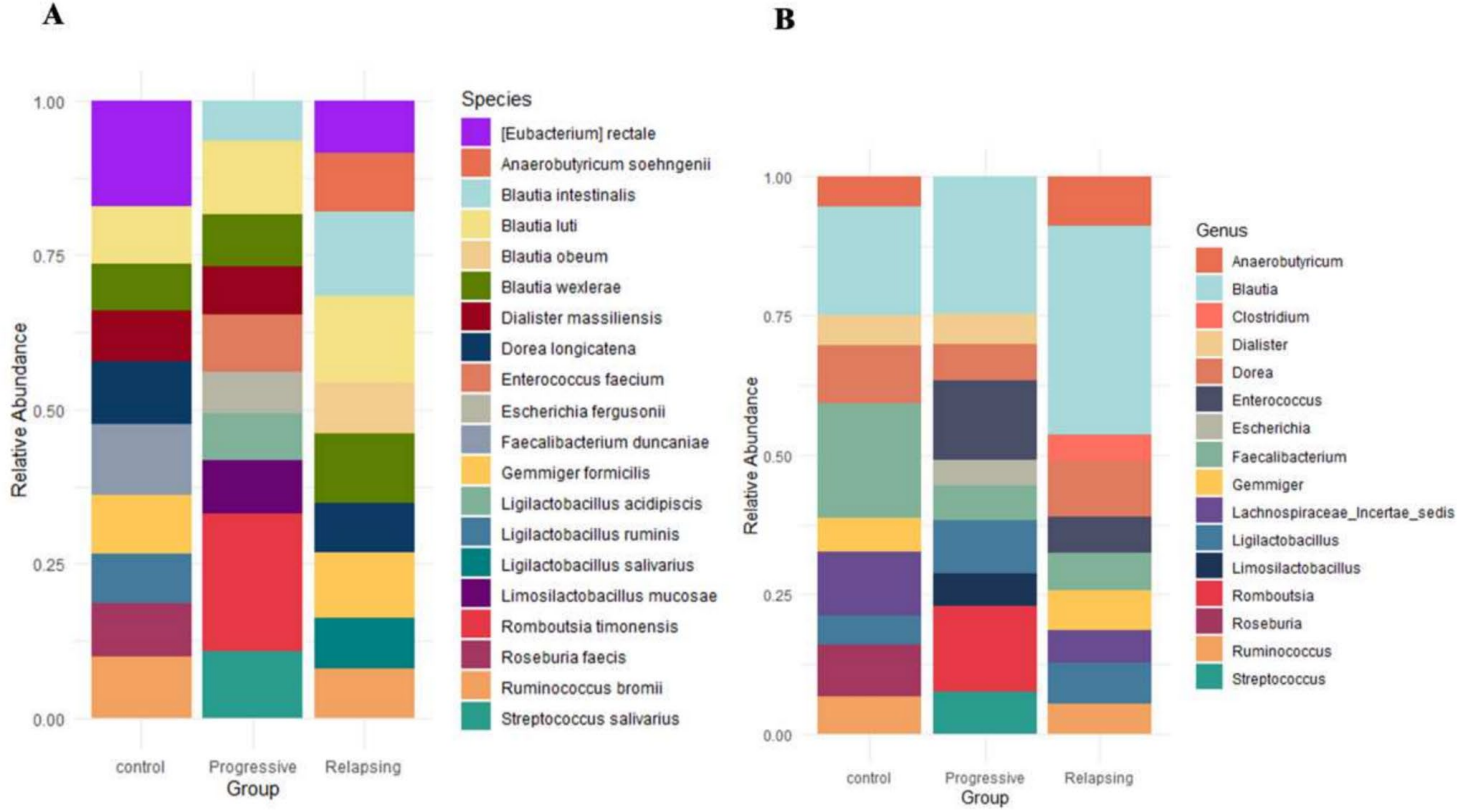
Relative abundance of prevalent microbiota (> 1% in any sample group) determined from Oxford Nanopore sequencing. Each bar represents the proportional contribution of various species (**A**) or genera (**B**) in each group with colors denoting specific taxa

*Blautia intestinalis* and *Blautia obeum* showed significant increases in the Relapsing group compared to the Control group, with rises of 241% (*P* = 0.003) and 228% (*P* = 0.02), respectively. These species also showed higher abundance in Relapsing versus Progressive MS (117% (*P* = 0.037) and 210% (*P* = 0.009), respectively), suggesting a role in inflammatory activity or relapse episodes. In contrast, *Romboutsia timonensis* and *Enterococcus faecium* were markedly enriched in the Progressive group relative to both the Control and Relapsing groups. Specifically, *Romboutsia timonensis* increased by 640% (*P* = 0.004) compared to Control and 244% (*P* = 0.017) compared to Relapsing MS, while *Enterococcus faecium* increased by 980% (*P* = 0.002) and 59% (*P* = 0.05), respectively. These trends suggest a potential link between these species and the neurodegenerative progression of MS (Fig. 6A).

Genus-level analyses revealed broader patterns, though interpretation is more complex due to species aggregation. For instance, the genus Blautia includes *Blautia intestinalis*, *Blautia obeum*, *Blautia wexlerae*, and *Blautia luti*, each potentially contributing differently to MS pathogenesis (Fig. 6B). This reinforces the importance of species-level analysis for precise functional interpretation.

**Fig. 7:**
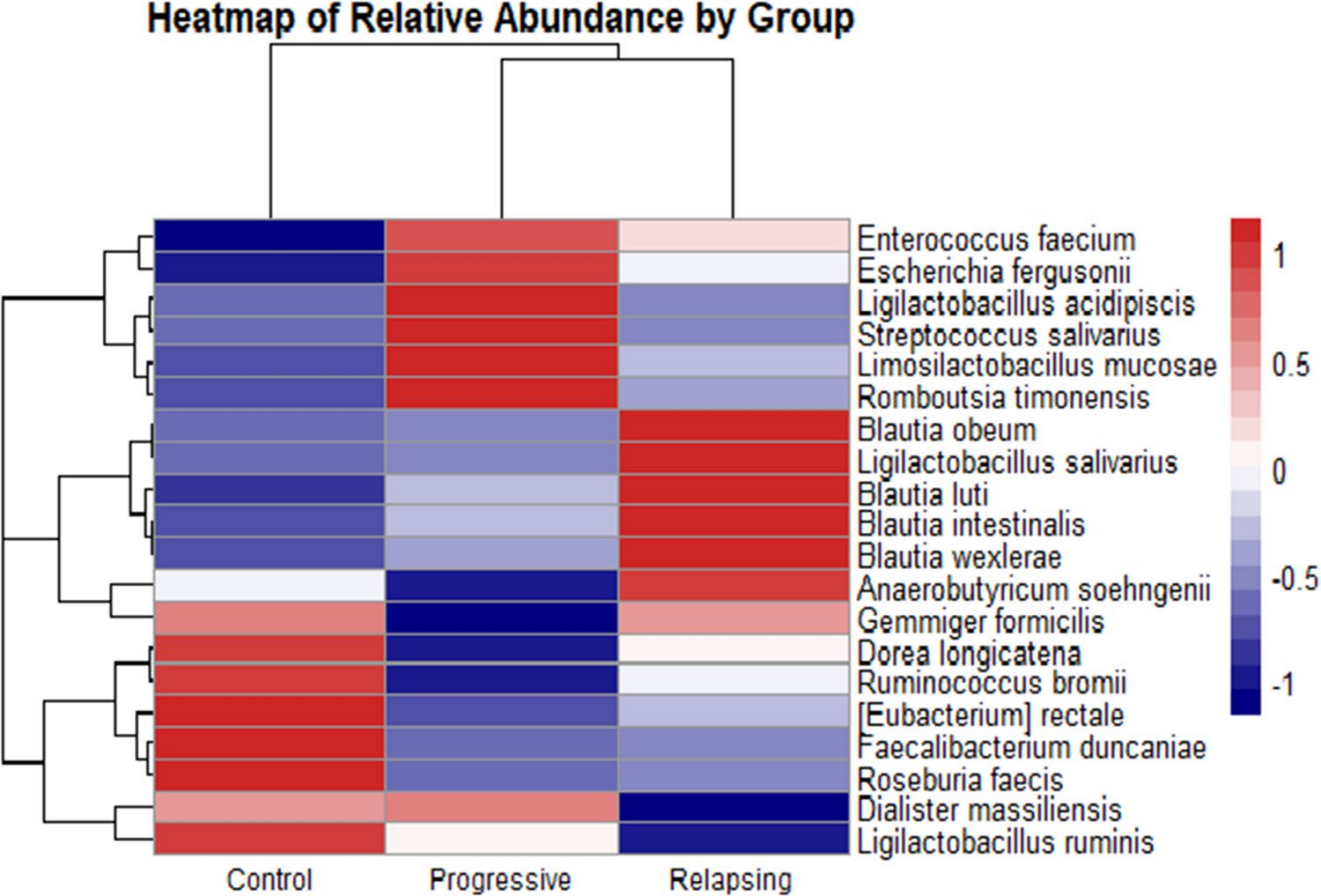
Heatmap of gut microbial relative abundance across groups

Heatmap clustering (Fig. 7) illustrated distinct microbial profiles for each group. The Control group was dominated by beneficial species such as *Roseburia faecis*, *Ruminococcus bromii*, *Eubacterium rectale*, *Faecalibacterium duncaniae*, *Dialister massiliensis*, and *Ligilactobacillus ruminis*. These taxa sharply declined in both MS subtypes, suggesting a loss of protective microbial populations. The Progressive MS group was defined by an overrepresentation of *Romboutsia timonensis*, *Enterococcus faecium*, *Streptococcus salivarius*, *Ligilactobacillus acidipiscis*, *Escherichia fergusonii*, and *Limosilactobacillus mucosae*. These bacteria exhibited a mean *z*-score of 1.327282 in the Progressive group, compared to − 0.978888 in the Control group and − 0.348394 in the Relapsing group, indicating a strong association with the progressive stage of MS. Meanwhile, the Relapsing group exhibited elevated levels of inflammation-associated species, including *Blautia intestinalis*, *Blautia obeum*, *Blautia luti*, *Gemmiger formicilis*, *Ligilactobacillus salivarius*, *Blautia wexlerae*, and *Anaerobutyricum soehngenii*. These species had a mean *z*-score of 1.270944 in the Relapsing group, compared to − 0.533267 in the Control and − 0.737677 in the Progressive groups, suggesting their potential role in relapse activity and inflammatory dysbiosis.

Within the top 30 most abundant species, *Clostridium saudiense* exhibited a highly significant rise in both Relapsing and Progressive MS. In contrast, beneficial SCFA-producing bacteria such as *Faecalibacterium butyricigenerans* and *Faecalibacterium duncaniae* were markedly depleted in the Relapsing group. *Enterococcus durans* and *Intestinibacter bartlettii* were enriched in Progressive MS, while *Gemmiger formicilis* declined significantly, further indicating dysbiosis.

**Fig. 8:**
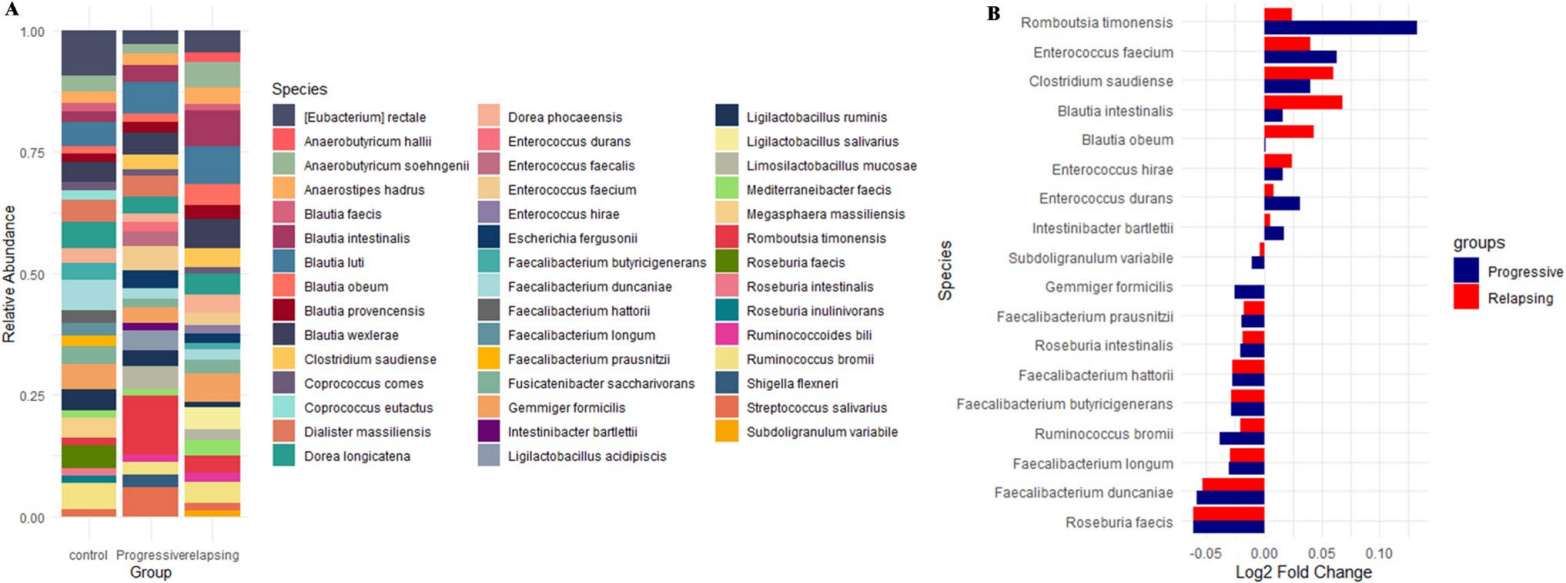
**A** Relative abundance of the top 30 prevalent microbiota species (> 1% in any sample group). The stacked bar plot illustrates microbial composition variations among the three groups. **B** Differential abundance analysis of microbial species between Progressive (blue) and Relapsing (red). Each species shown has a statistically significant difference (*P* < 0.05), even if not among the top 30 most abundant species. The Log2 fold change highlights species with increased or decreased abundance between the two groups

*Ruminococcus bromii* and *Faecalibacterium duncaniae* were both significantly reduced in the Progressive group, reinforcing the loss of commensal species. Additionally, multiple beneficial species common in healthy controls including *Faecalibacterium longum*, *Faecalibacterium prausnitzii*, *Roseburia faecis*, *Faecalibacterium hattorii*, and *Roseburia intestinalis* were significantly reduced in both Relapsing and Progressive MS groups (Fig. 8A, B).

Collectively, these findings highlight a clear shift in microbial community structure associated with MS, with subtype-specific dysbiosis patterns. The Progressive group is characterized by dominance of proinflammatory taxa and depletion of beneficial microbes, while the Relapsing group exhibits elevated inflammation-associated species with partial preservation of protective taxa. These observations underscore potential microbial biomarkers and targets for therapeutic intervention in MS.

Each row represents a bacterial species, and each column represents a participant group (Control, Progressive MS, Relapsing MS). The color gradient reflects *z*-score normalized relative abundance values, where red denotes higher-than-average abundance, and blue denotes lower-than-average abundance for each species across samples. The heatmap highlights distinct microbial signatures in MS. Progressive MS is associated with a severe dysbiotic shift, characterized by an increase in inflammation-associated species and a marked reduction in health-associated microbes. Relapsing MS exhibits intermediate dysbiosis, with a partial loss of beneficial species and moderate increases in pro-inflammatory bacteria. In contrast, the Control group displays a balanced gut microbiota, dominated by beneficial, health-associated species.

### Functional Enrichment Analysis

**Fig. 9:**
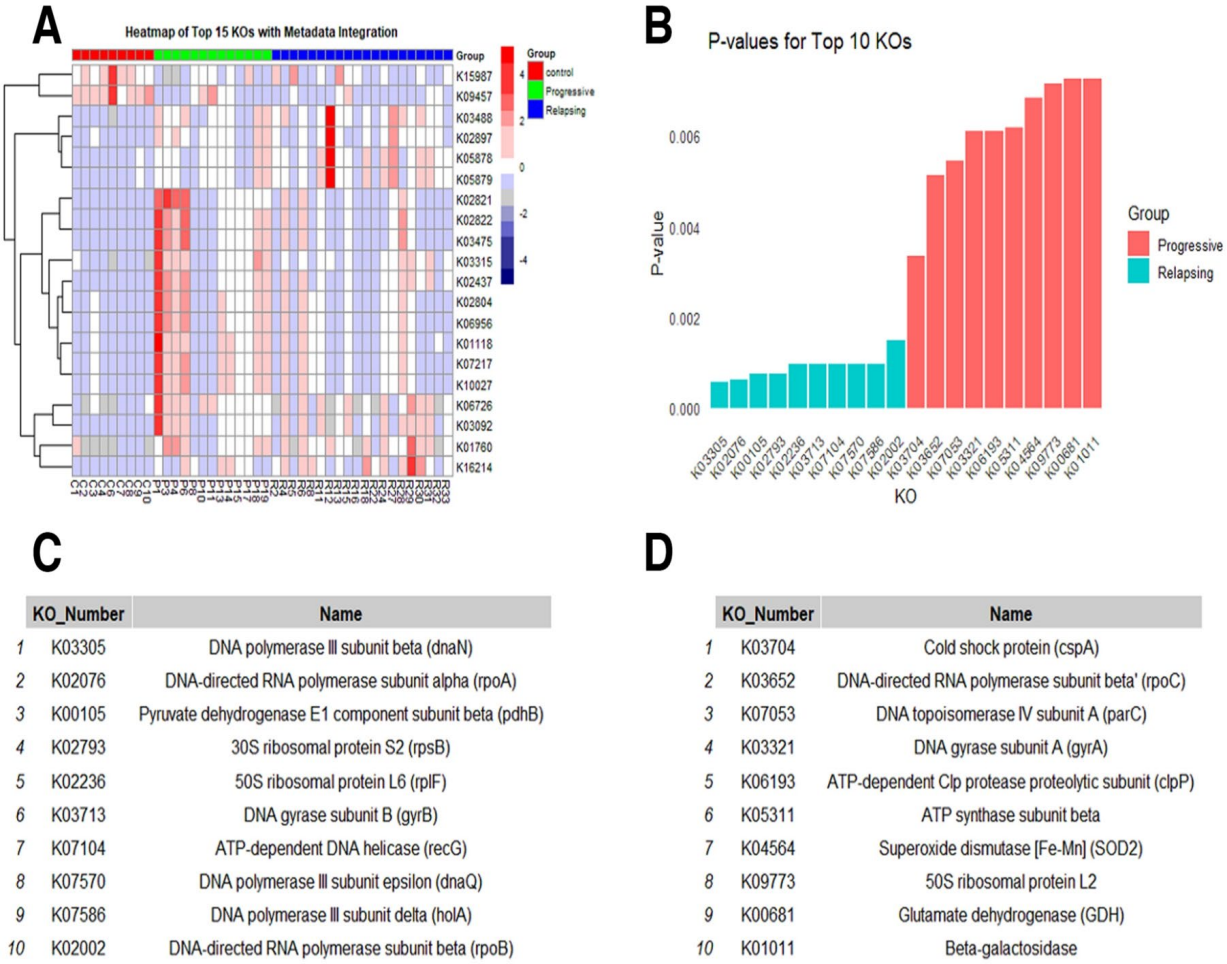
**A** Heatmap showing the scaled abundance of the top 15 KOs across Control, Progressive MS, and Relapsing MS groups. The rows represent individual KOs, while the columns denote individual samples. The color intensity indicates the scaled abundance of each KO, with red representing higher abundance and blue representing lower abundance. **B** Bar plot illustrating the *P*-values for the top 10 significant KOs, comparing Progressive MS (red) and Relapsing MS (blue) to the control group. **C** KO_Name_in Progressive. **D** KO_Name_in Relapsing

PICRUSt2 was used to infer the functional content of the microbiota based on closed-reference OTUs. The heatmap (Fig. 9A) illustrates the relative abundance of the top 15 KEGG Orthologs (KOs) across groups. KOs such as K15987 and K09457 were predominantly enriched in the control group, suggesting a potential protective role. In contrast, K03488 and K02897 showed significant enrichment in Progressive MS samples, indicating possible involvement in disease progression mechanisms. Additionally, K03092 and K16214 were notably enriched in the Relapsing MS group.

The bar graph (Fig. 9B) displays the statistical significance (*P*-values) of the top 10 KOs for both the Progressive MS (red) and Relapsing MS (blue) groups compared to the control group. KOs such as K03704 and K03652 showed the most significant differences in the Progressive group, with *P*-values below 0.002 and 0.005, respectively. Similarly, highly significant *P*-values were observed for K03305 and K02076 in the Relapsing group. Most of the significant KOs in the Progressive group are linked to pathways involved in chronic inflammation and metabolic dysregulation, while those in the Relapsing group are associated with immune response regulation and microbial metabolism. A full description of each KO is provided in Fig. 9C, D.

### Predicted Metabolic Pathway of Bacterial Communities

**Fig. 10:**
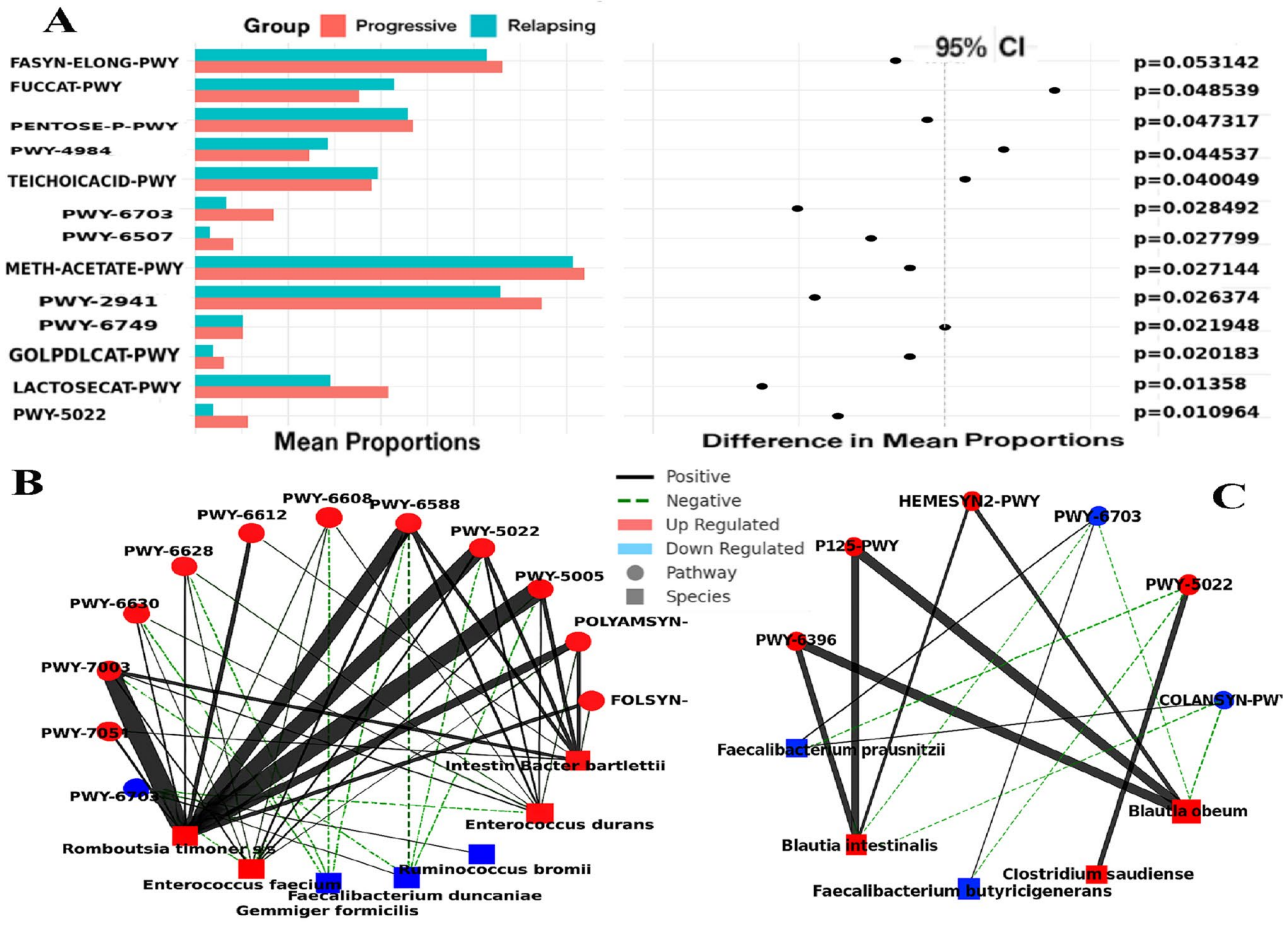
**A** The mean proportions and differences in microbial composition between the Progressive and Relapsing groups compared to the Control group, with 95% confidence intervals indicating statistical significance. **B** Species-pathway correlation network in the Progressive MS Group. Edge thickness represents the strength of the correlation, with thicker edges indicating stronger correlations. Solid black lines = positive correlations, dashed green lines = negative correlations. **C** Species-pathway correlation network in the Relapsing MS group. Similarly, edge thickness represents the strength of the correlation, with thicker edges indicating stronger correlations. Solid black lines = positive correlations, dashed green lines = negative correlations

The control group served as a baseline to assess deviations in microbial metabolic activity. Both the Progressive and Relapsing MS groups exhibited significant shifts in specific pathways compared to the control group. Figure 10 presents a two-panel visualization showing the mean proportions and differences in mean proportions (with 95% confidence intervals) for key pathways in the Progressive and Relapsing groups relative to the control group. In the left panel, differences in mean proportions are represented by black dots with corresponding confidence intervals. Values to the right of the dashed line indicate higher proportions in the Relapsing group, while values to the left indicate higher proportions in the Progressive group. *P*-values indicating statistical significance are shown on the far right (Fig. 10A).

The METH-ACETATE-PWY, PWY-2941, and FASYN-ELONG-PWY pathways were significantly increased in both MS groups. In contrast, PWY-6749 and PWY-6703 showed a decrease in both groups. Notably, PWY-6703 exhibited a more pronounced reduction in the Relapsing group, with a 1.3-fold decrease compared to the Progressive group, suggesting its potential importance in the relapsing form of the disease. On the other hand, PWY-5022 was elevated in the Progressive group relative to the control group, showing a 1.5-fold increase compared to the Relapsing group. These findings indicate dynamic regulation of this pathway across different stages of MS.

The network illustrates statistically significant correlations (*P* < 0.05) between bacterial species (square nodes) and metabolic pathways (circular nodes). Red nodes represent upregulated species or pathways, while blue nodes represent downregulated ones. Solid black lines indicate positive correlations, whereas dashed black lines indicate negative correlations. This visualization highlights the functional interactions between gut microbiota and metabolic pathways in relation to the Relapsing and Progressive subtypes of MS.

In Progressive MS, the upregulated pathways—FOLSYN-PWY, POLYAMSYN-PWY, PWY-5005, PWY-5022, PWY-6588, PWY-6608, PWY-6612, PWY-6628, PWY-6630, PWY-7003, and PWY-7254—are associated with immune modulation and neuroinflammation. These pathways show positive correlations with upregulated species such as *Romboutsia timonensis*, *Enterococcus faecium*, and *Intestinibacter bartlettii*, which may contribute to chronic inflammation. Conversely, negative correlations were observed with *Gemmiger formicilis* and *Faecalibacterium duncaniae*, both downregulated species known for their immunoregulatory roles. Their depletion may promote neuroinflammation and accelerate disease progression in Progressive MS (Fig. 10B).

In Relapsing MS, upregulated pathways HEMESYN2-PWY, PWY-5022, and P125-PWY are positively correlated with species such as *Blautia intestinalis* and *Blautia obeum*, which are involved in immune modulation and maintenance of intestinal barrier integrity. Meanwhile, the downregulated pathways COLANSYN-PWY and PWY-6396 are associated with *Faecalibacterium duncaniae*, a species recognized for its anti-inflammatory properties (Fig. 10C).

## Discussion

### Microbial Diversity and Community Structure

In this study, we employed long-read sequencing to investigate the gut microbiome composition and functional potential in Egyptian patients with multiple sclerosis (MS), including both relapsing and progressive subtypes, compared to healthy controls. Our analysis of microbial diversity indices revealed that MS patients exhibit increased species richness but decreased evenness, suggesting a microbial imbalance driven by the overrepresentation of specific taxa. These findings are consistent with those of Ghimire et al. (2025) [24], who also observed increased richness in MS but with skewed distribution, implying dysbiosis. In contrast, other studies such as Miyake et al. (2015) observed a decline in overall α-diversity in MS, underscoring the heterogeneous and population-specific nature of microbial alterations associated with the disease [25]. This inconsistency may also be attributed to methodological differences, including sequencing platforms, target regions, and MS subtype representation. Miyake et al. used 16S rRNA gene sequencing targeting the V1–V2 regions on the now-outdated Roche 454 pyrosequencing platform, which provides limited taxonomic resolution and depth. In contrast, Ghimire et al. employed metagenomic shotgun sequencing (2 × 100 bp) using the DNBSEQ-G400 platform, offering higher resolution and a broader functional profile. Beyond methodological factors, regional dietary habits—such as higher intake of fiber, legumes, and fermented foods in Egyptian diets—and climatic factors like increased sun exposure, which may influence vitamin D levels, could further impact gut microbiota composition. These region-specific influences likely contribute to the distinct microbial patterns observed in our cohort and emphasize the need for geographically diverse microbiome studies in MS. Similarly, studies from other non-Western populations—such as those conducted in Iran, India, and the broader Middle East—have reported microbial alterations that partially overlap with our findings. These regions share comparable environmental exposures, including higher sunlight levels that influence vitamin D metabolism, and traditional dietary patterns rich in fiber, legumes, and fermented foods. Such similarities may support the presence or preservation of SCFA-producing taxa and modulate immune responses differently than in Westernized settings. For example, *Faecalibacterium prausnitzii* and *Roseburia* species were relatively preserved in Indian MS cohorts, aligning with trends observed in our Egyptian control group. In Iranian patients, a reduction in *Prevotella* and enrichment of pro-inflammatory taxa like *Escherichia* were reported [26], patterns that closely resemble those in our Progressive MS group. These cross-regional parallels highlight the importance of considering geographic and lifestyle-related factors such as diet and sun exposure when interpreting microbiome shifts in MS across diverse populations.

β-Diversity analysis further confirmed significant structural differences among the Control, Relapsing MS, and Progressive MS groups. Distinct clustering patterns were evident through UniFrac-based analyses, with both weighted and unweighted metrics revealing differences in taxa abundance and composition. These findings are consistent with studies by Jangi et al. (2016) and Berer et al. (2017) [27] [6], which highlighted microbiota shifts associated with MS progression. The partial overlap observed between the Relapsing and Progressive groups suggests shared dysbiotic features contributing to MS pathogenesis.

### Species-Level Findings and Microbial Signatures

Taxonomic profiling revealed distinct compositional changes in gut microbiota across the three groups. The Control group was enriched in beneficial taxa, such as *Faecalibacterium longum*, *Roseburia intestinalis*, *Faecalibacterium hattorii*, *Roseburia faecis*, and *Faecalibacterium prausnitzii*. These species are known for maintaining gut barrier integrity, modulating immune responses, and producing short-chain fatty acids (SCFAs) like butyrate [28]. Their reduction in both MS subtypes suggests a loss of protective microbial functions.

In the Progressive group, we observed increased abundance of pro-inflammatory species such as *Enterococcus faecium*, *Romboutsia timonensis*, *Enterococcus durans*, and *Intestinibacter bartlettii*, indicating a shift toward a pro-inflammatory gut environment. *Enterococcus faecium*, for example, is known to promote IL-6 and TNF-α production and has been linked to gut permeability and systemic inflammation [29]. It is also elevated in inflammatory bowel disease (IBD), a condition that shares immunopathological features with MS [30]. Likewise, *Enterococcus durans* can generate reactive oxygen species (ROS), potentially exacerbating oxidative stress and neuroinflammation.

In contrast, several beneficial bacteria known for their anti-inflammatory and SCFA-producing roles were significantly reduced in the Progressive MS group. These included *Ruminococcus bromii*, a keystone species for fiber degradation and butyrate production; *Gemmiger formicilis*, involved in immune modulation; and *Faecalibacterium duncaniae*, a commensal associated with regulatory immune functions. The depletion of *Ruminococcus bromii* may impair gut barrier function and intensify inflammation [31]. Notably, both *Faecalibacterium butyricigenerans* and *Faecalibacterium duncaniae* were reduced in both Relapsing and Progressive MS, suggesting compromised microbial regulation of inflammation. This compositional shift reflects a dysbiotic state characterized by a loss of SCFA producers, potentially leading to decreased butyrate, acetate, and propionate levels. These SCFAs are essential for maintaining gut barrier integrity, regulating immune responses, and suppressing neuroinflammation. Such microbial depletion may weaken gut-immune signaling and contribute to increased disease activity and progression [32] Ling et al. (2020) reported that reduced SCFA levels were strongly associated with neuroinflammation in MS. Therefore, this microbial depletion may exacerbate disease severity by weakening gut-immune signaling and promoting inflammation [33].

### Functional Interpretation: Metabolic and Pathway Shifts

Using PICRUSt2, we identified significant functional differences in microbial communities between MS subtypes. To enhance interpretability, these changes were organized into key biological themes. In Progressive MS, we observed upregulation of several oxidative stress-related genes, including superoxide dismutase (K04564) and glutamate dehydrogenase (K00681), both of which are known to promote reactive oxygen species (ROS) production and may exacerbate neuroinflammation [34] [35]. Additionally, *ATP-*dependent Clp protease (K06193), a gene implicated in bacterial stress responses, may further disrupt host-microbiota signaling along the gut-brain axis.

Markers of increased transcriptional and metabolic activity were also elevated in Progressive MS. DNA gyrase (K03321) and 50S ribosomal protein L2 (K09773) suggest enhanced bacterial replication and protein synthesis, potentially elevating antigenic stimulation of the host immune system. Upregulation of beta-galactosidase (K01011), which is involved in carbohydrate metabolism, may contribute to altered gut metabolic profiles and increased mucosal inflammation [36].

Several inflammatory and immune-modulatory pathways, including FOLSYN-PWY, POLYAMSYN-PWY, PWY-5005, and PWY-5022, were enriched in Progressive MS. These pathways were primarily driven by pro-inflammatory species such as *Romboutsia timonensis* and *Enterococcus faecium*, both of which are linked to cytokine activation and systemic inflammation. In contrast, the protective metabolic pathway PWY-6703, which is associated with metabolic resilience, was downregulated, potentially impairing homeostatic functions and accelerating disease progression [37].

In Relapsing MS, there was evidence of mitochondrial dysfunction and genomic instability, as indicated by the altered expression of genes such as pyruvate dehydrogenase (K00105), DNA polymerase subunits (K03305, K07570), and RNA helicases (K07104). These disruptions are increasingly recognized as contributors to neurodegenerative processes in MS [38] [39]. Furthermore, pathways associated with immune modulation and oxidative stress, including HEMESYN2-PWY, PWY-6396, and P125-PWY, were enriched, whereas COLANSYN-PWY and PWY-6703 were downregulated, suggesting reduced microbial capacity for metabolic adaptation and immune tolerance.

Notably, the inflammatory pathway PWY-5022 showed strong positive correlations with *Blautia obeum*, *Blautia intestinalis*, and *Clostridium saudiense*, linking specific microbial taxa to immune dysregulation and increased relapse risk. These observations underscore the functional divergence between Progressive and Relapsing MS and highlight the potential of microbial metabolic pathways as biomarkers and therapeutic targets.

### Clinical Relevance and Implications

Our cohort included 43 individuals (10 controls, 20 Relapsing MS, and 13 Progressive MS), with 67% females reflecting the known gender bias in MS incidence. Matched age and BMI ranges (24–26) to minimize confounders such as age-related microbial variation and obesity-associated dysbiosis. Correlations between microbial shifts and clinical indicators such as EDSS scores, total relapses, and disease duration suggest that microbiota composition could serve as a non-invasive biomarker for disease monitoring.

Montgomery et al. (2024) [40] demonstrated that gut microbiota signatures predict MS progression, while Cox et al. (2021) [34] reported associations between microbial taxa and disability severity. Our results reinforce these findings: *Clostridium saudiense* and *Enterococcus faecium* correlated with higher EDSS scores, while *Ruminococcus bromii* abundance was linked to lower scores, implying a protective effect. This supports the role of specific bacteria in modulating disease severity.

Additionally, reduced SCFA production may diminish Treg function. Duscha et al. (2020) [41] showed that propionate supplementation in MS enhances Treg activity, highlighting the clinical relevance of maintaining SCFA-producing microbiota. Thus, targeting microbial metabolites or restoring beneficial taxa may offer new therapeutic avenues. The inclusion of family members as controls may introduce some degree of genetic or environmental similarity. However, this was purposefully done to replicate shared lifestyle factors such as diet, housing, and geographic exposure, thereby controlling for potential lifestyle-related confounders in gut microbiota composition.

Our findings highlight specific microbial taxa and functional pathways that differ between MS subtypes, suggesting potential clinical utility in disease monitoring and management. Notably, *Enterococcus faecium*, *Romboutsia timonensis*, and *Clostridium saudiense* were enriched in both RMS and PMS patients and were associated with pro-inflammatory profiles, whereas beneficial SCFA-producing bacteria such as *Faecalibacterium duncaniae* and *Ruminococcus bromii* were consistently depleted. These shifts in microbial composition may reflect disease-specific immune dysregulation and neuroinflammatory states and thus could serve as non-invasive biomarkers to stratify MS subtypes or monitor disease progression. Furthermore, functional prediction analyses using PICRUSt2 revealed altered metabolic signatures in PMS, including pathways related to oxidative stress and immune dysfunction, such as increased expression of enzymes like superoxide dismutase and glutamate dehydrogenase. These findings support the potential for microbiota-informed clinical strategies, including personalized dietary modulation, probiotic or postbiotic interventions, or adjunct therapies aimed at restoring SCFA-producing communities and mitigating inflammation in progressive MS. Ultimately, our study contributes to the growing foundation for microbiota-targeted approaches in MS care, particularly for patients with rapid progression or limited response to standard immunotherapies.

Despite these insights, a key limitation of this study is the relatively small sample size, which may reduce the statistical power to detect subtle microbiota differences and limits the generalizability of the findings to the broader MS population. Future studies with larger and more diverse cohorts are warranted to validate and extend these observations.

## Conclusion

This study highlights profound alterations in microbial diversity, composition, and function in MS, with distinct patterns in Progressive and Relapsing subtypes. Progressive MS is marked by pro-inflammatory taxa, functional enrichment of oxidative stress pathways, and loss of SCFA producers supporting a neurodegenerative trajectory. Relapsing MS shows a mixed profile, with both inflammatory and compensatory anti-inflammatory features. These findings offer valuable insights into the gut-immune-CNS axis in MS and support microbiota-based strategies for diagnosis and intervention.

## Data Availability

The datasets generated or analysed during the current study are not publicly available but are available from the corresponding author on reasonable request.

## References

[1] Papiri G, D’Andreamatteo G, Cacchiò G et al (2023) Multiple sclerosis: inflammatory and neuroglial aspects. Curr Issues Mol Biol 45:1443–1470 36826039 10.3390/cimb45020094 PMC9954863

[2] Perrone V, Veronesi C, Giacomini E et al (2022) The epidemiology, treatment patterns and economic burden of different phenotypes of multiple sclerosis in Italy: relapsing-remitting multiple sclerosis and secondary progressive multiple sclerosis. Clin Epidemiol 14:1327–1337. 10.2147/CLEP.S376005 36387930 10.2147/CLEP.S376005 PMC9648183

[3] Walton C, King R, Rechtman L et al (2020) Rising prevalence of multiple sclerosis worldwide: insights from the Atlas of MS, third edition. Multi Sclerosis J 26:1816–1821. 10.1177/1352458520970841 10.1177/1352458520970841 PMC7720355 33174475

[4] Portaccio E, Magyari M, Havrdova EK et al (2024) Multiple sclerosis: emerging epidemiological trends and redefining the clinical course. The Lancet Reg Health - Eur 44(3):100977. 10.1016/j.lanepe.2024.100977 39444703 10.1016/j.lanepe.2024.100977 PMC11496978

[5] Cekanaviciute E, Yoo BB, Runia TF et al (2017) Gut bacteria from multiple sclerosis patients modulate human T cells and exacerbate symptoms in mouse models. Proc Natl Acad Sci USA 114(40):10713–10718. 10.1073/pnas.1711235114 28893978 10.1073/pnas.1711235114 PMC5635915

[6] Berer K, Gerdes LA, Cekanaviciute E et al (2017) Gut microbiota from multiple sclerosis patients enables spontaneous autoimmune encephalomyelitis in mice. Proc Natl Acad Sci USA 114(40):10719–10724. 10.1073/pnas.1711233114 28893994 10.1073/pnas.1711233114 PMC5635914

[7] Ochoa-Repáraz J, Kirby TO, Kasper LH (2018) The gut microbiome and multiple sclerosis. Cold Spring Harb Perspect Med 8:. 10.1101/cshperspect.a029017 10.1101/cshperspect.a029017 PMC5983160 29311123

[8] Wang X, Liang Z, Wang S et al (2021) Role of gut microbiota in multiple sclerosis and potential therapeutic implications. Curr Neuropharmacol 20:1413–1426. 10.2174/1570159x19666210629145351 10.2174/1570159X19666210629145351 PMC9881072 34191698

[9] Camara-Lemarroy CR, Metz LM, Yong VW (2018) Focus on the gut-brain axis: multiple sclerosis, the intestinal barrier and the microbiome. World J Gastroenterol 24:4217–4223 30310254 10.3748/wjg.v24.i37.4217 PMC6175760

[10] Al KF, Craven LJ, Gibbons S, et al (2022) Fecal microbiota transplantation is safe and tolerable in patients with multiple sclerosis: a pilot randomized controlled trial. Mult Scler J Exp Transl Clin 8:. 10.1177/20552173221086662 10.1177/20552173221086662 PMC9102167 35571974

[11] Waubant E, Lucas R, Mowry E et al (2019) Environmental and genetic risk factors for MS: an integrated review. Ann Clin Transl Neurol 6:1905–1922 31392849 10.1002/acn3.50862 PMC6764632

[12] Johnson JS, Spakowicz DJ, Hong BY, et al (2019) Evaluation of 16S rRNA gene sequencing for species and strain-level microbiome analysis. Nat Commun 10:. 10.1038/s41467-019-13036-1 10.1038/s41467-019-13036-1 PMC6834636 31695033

[13] Edgar RC (2018) Updating the 97% identity threshold for 16S ribosomal RNA OTUs. Bioinformatics 34:2371–2375. 10.1093/bioinformatics/bty113 29506021 10.1093/bioinformatics/bty113

[14] Benítez-Páez A, Portune KJ, Sanz Y (2016) Species-level resolution of 16S rRNA gene amplicons sequenced through the MinIONTM portable nanopore sequencer. Gigascience 5:. 10.1186/s13742-016-0111-z 10.1186/s13742-016-0111-z PMC4730766 26823973

[15] Curry KD, Wang Q, Nute MG et al (2022) Emu: species-level microbial community profiling of full-length 16S rRNA Oxford Nanopore sequencing data. Nat Methods 19:845–853. 10.1038/s41592-022-01520-4 35773532 10.1038/s41592-022-01520-4 PMC9939874

[16] De Coster W, D’Hert S, Schultz DT et al (2018) NanoPack: visualizing and processing long-read sequencing data. Bioinformatics 34:2666–2669. 10.1093/bioinformatics/bty149 29547981 10.1093/bioinformatics/bty149 PMC6061794

[17] Li H (2018) Minimap2: pairwise alignment for nucleotide sequences. Bioinformatics 34:3094–3100. 10.1093/bioinformatics/bty191 29750242 10.1093/bioinformatics/bty191 PMC6137996

[18] Li H, Handsaker B, Wysoker A et al (2009) The sequence alignment/map format and SAMtools. Bioinformatics 25:2078–2079. 10.1093/bioinformatics/btp352 19505943 10.1093/bioinformatics/btp352 PMC2723002

[19] Shen W, Xiong J (2019) TaxonKit: a cross-platform and efficient NCBI taxonomy toolkit. J Genet Genomics 48(9):844–850. 10.1016/j.jgg.2021.03.006 10.1016/j.jgg.2021.03.006 34001434

[20] Lee C, Patil S, Sartor MA (2016) RNA-Enrich: a cut-off free functional enrichment testing method for RNA-seq with improved detection power. Bioinformatics 32:1100–1102. 10.1093/bioinformatics/btv694 26607492 10.1093/bioinformatics/btv694 PMC5860544

[21] McMurdie PJ, Holmes S (2013) Phyloseq: an R package for reproducible interactive analysis and graphics of microbiome census data. PLoS One 8:. 10.1371/journal.pone.0061217 10.1371/journal.pone.0061217 PMC3632530 23630581

[22] Caicedo HH, Hashimoto DA, Caicedo JC et al (2020) Overcoming barriers to early disease intervention. Nat Biotechnol 38:669–673 32444852 10.1038/s41587-020-0550-z

[23] Lozupone C, Lladser ME, Knights D et al (2011) UniFrac: an effective distance metric for microbial community comparison. ISME J 5:169–172 20827291 10.1038/ismej.2010.133 PMC3105689

[24] Ghimire S, Lehman PC, Aguilar Meza LS, et al (2025) Specific microbial ratio in the gut microbiome is associated with multiple sclerosis. Proc Natl Acad Sci U S A 122:. 10.1073/pnas.2413953122 10.1073/pnas.2413953122 PMC11912405 40030030

[25] Miyake S, Kim S, Suda W, et al (2015) Dysbiosis in the gut microbiota of patients with multiple sclerosis, with a striking depletion of species belonging to clostridia XIVa and IV clusters. PLoS One 10:. 10.1371/journal.pone.0137429 10.1371/journal.pone.0137429 PMC4569432 26367776

[26] Chen J, Chia N, Kalari KR, et al (2016) Multiple sclerosis patients have a distinct gut microbiota compared to healthy controls. Sci Rep 6:. 10.1038/srep28484 10.1038/srep28484 PMC4921909 27346372

[27] Jangi S, Gandhi R, Cox LM, et al (2016) Alterations of the human gut microbiome in multiple sclerosis. Nat Commun 7:. 10.1038/ncomms12015 10.1038/ncomms12015 PMC4931233 27352007

[28] Maaskant A, Voermans B, Levin E, et al (2024) Microbiome signature suggestive of lactose-intolerance in rhesus macaques (Macaca mulatta) with intermittent chronic diarrhea. Anim Microbiome 6:. 10.1186/s42523-024-00338-z 10.1186/s42523-024-00338-z PMC11421201 39313845

[29] Pröbstel AK, Zhou X, Baumann R, et al (2020) Gut microbiota–specific iga+ B cells traffic to the CNS in active multiple sclerosis. Sci Immunol 5:. 10.1126/SCIIMMUNOL.ABC7191 10.1126/sciimmunol.abc7191 PMC8043673 33219152

[30] Schepici G, Silvestro S, Bramanti P, Mazzon E (2019) The gut microbiota in multiple sclerosis: an overview of clinical trials. Cell Transplant 28:1507–1527 31512505 10.1177/0963689719873890 PMC6923550

[31] Li J, Feng S, Wang Z, et al (2023) Limosilactobacillus mucosae-derived extracellular vesicles modulates macrophage phenotype and orchestrates gut homeostasis in a diarrheal piglet model. NPJ Biofilms Microbiomes 9:. 10.1038/s41522-023-00403-6 10.1038/s41522-023-00403-6 PMC10244441 37280255

[32] Ling Z, Cheng Y, Yan X, et al (2020) Alterations of the fecal microbiota in Chinese patients with multiple sclerosis. Front Immunol 11:. 10.3389/fimmu.2020.590783 10.3389/fimmu.2020.590783 PMC7772405 33391265

[33] Ordoñez-Rodriguez A, Roman P, Rueda-Ruzafa L et al (2023) Changes in gut microbiota and multiple sclerosis: a systematic review. Int J Environ Res Public Health 20(5):4624. 10.3390/ijerph20054624 36901634 10.3390/ijerph20054624 PMC10001679

[34] Cox LM, Maghzi AH, Liu S et al (2021) Gut microbiome in progressive multiple sclerosis. Ann Neurol 89:1195–1211. 10.1002/ana.26084 33876477 10.1002/ana.26084 PMC8132291

[35] Werner P, Pitt D, Raine CS (2001) Multiple sclerosis: altered glutamate homeostasis in lesions correlates with oligodendrocyre and axonal damage. Ann Neurol 50:169–180. 10.1002/ana.1077 11506399 10.1002/ana.1077

[36] Thirion F, Sellebjerg F, Fan Y, et al (2023) The gut microbiota in multiple sclerosis varies with disease activity. Genome Med 15:. 10.1186/s13073-022-01148-1 10.1186/s13073-022-01148-1 PMC9814178 36604748

[37] Cekanaviciute E, Pröbstel A-K, Thomann A et al (2018) Multiple sclerosis-associated changes in the composition and immune functions of spore-forming bacteria. mSystems 3(6):e00083-18. 10.1128/mSystems.00083-18 30417113 10.1128/mSystems.00083-18 PMC6222044

[38] Campbell G, Mahad DJ (2018) Mitochondrial dysfunction and axon degeneration in progressive multiple sclerosis. FEBS Lett 592:1113–1121 29453889 10.1002/1873-3468.13013

[39] Tiwari V, Wilson DM (2019) DNA damage and associated DNA repair defects in disease and premature aging. Am J Hum Genet 105:237–257 31374202 10.1016/j.ajhg.2019.06.005 PMC6693886

[40] Montgomery TL, Wang Q, Mirza A, et al (2024) Identification of commensal gut microbiota signatures as predictors of clinical severity and disease progression in multiple sclerosis. Sci Rep 14:. 10.1038/s41598-024-64369-x 10.1038/s41598-024-64369-x PMC11222390 38961134

[41] Duscha A, Gisevius B, Hirschberg S et al (2020) Propionic acid shapes the multiple sclerosis disease course by an immunomodulatory mechanism. Cell 180:1067-1080.e16. 10.1016/j.cell.2020.02.035 32160527 10.1016/j.cell.2020.02.035

